# Willingness of Men Who Have Sex with Men (MSM) in the United States to Be Circumcised as Adults to Reduce the Risk of HIV Infection

**DOI:** 10.1371/journal.pone.0002731

**Published:** 2008-07-16

**Authors:** Elin B. Begley, Krishna Jafa, Andrew C. Voetsch, James D. Heffelfinger, Craig B. Borkowf, Patrick S. Sullivan

**Affiliations:** Division of HIV/AIDS Prevention, National Center for HIV/AIDS, Viral Hepatitis, STD, and TB Prevention, Centers for Disease Control and Prevention, Atlanta, Georgia, United States of America; University of Cape Town, South Africa

## Abstract

**Background:**

Circumcision reduces HIV acquisition among heterosexual men in Africa, but it is unclear if circumcision may reduce HIV acquisition among men who have sex with men (MSM) in the United States, or whether MSM would be willing to be circumcised if recommended.

**Methods:**

We interviewed presumed-HIV negative MSM at gay pride events in 2006. We asked uncircumcised respondents about willingness to be circumcised if it were proven to reduce risk of HIV among MSM and perceived barriers to circumcision. Multivariate logistic regression was used to identify covariates associated with willingness to be circumcised.

**Results:**

Of 780 MSM, 133 (17%) were uncircumcised. Of these, 71 (53%) were willing to be circumcised. Willingness was associated with black race (exact odds ratio [OR]: 3.4, 95% confidence interval [CI]: 1.3–9.8), non-injection drug use (OR: 6.1, 95% CI: 1.8–23.7) and perceived reduced risk of penile cancer (OR: 4.7, 95% CI: 2.0–11.9). The most commonly endorsed concerns about circumcision were post-surgical pain and wound infection.

**Conclusions:**

Over half of uncircumcised MSM, especially black MSM, expressed willingness to be circumcised. Perceived risks and benefits of circumcision should be a part of educational materials if circumcision is recommended for MSM in the United States.

## Introduction

Circumcision has recently been shown to be effective in decreasing HIV acquisition among adult heterosexual males in several sub-Saharan African countries. Recent clinical trials in South Africa, Kenya and Uganda showed that adult circumcision reduced HIV acquisition by 50–60%.[1], [2], [3] As a result, the World Health Organization currently recommends male circumcision as part of a comprehensive HIV prevention approach in Africa.^4^ However, these trials do not provide direct evidence that circumcision decreases HIV acquisition among men who engage in insertive or receptive anal sex. In the United States, the primary mode of HIV transmission is male-male sex.[4] One prospective cohort study in the U.S. demonstrated a reduced risk of HIV infection among circumcised men who have sex with men (MSM) compared with uncircumcised MSM, [5] suggesting that circumcision may decrease HIV transmission among MSM. In addition, another study found a trend toward a protective effect of circumcision against syphilis infection among heterosexual males in the United States.[6] In contrast, a study using respondent-driven sampling showed no association between circumcision status and HIV seroconversion in black and Latino MSM.[7]

Even if circumcision is shown to be an effective means of preventing HIV infection among MSM, it is not known if adult MSM in the United States would be willing to be circumcised. A meta-analysis of thirteen studies in sub-Saharan Africa demonstrated that 65% of uncircumcised men reported that they would be willing to be circumcised, indicating that this might be a widely acceptable HIV prevention method among heterosexual men in this region.[8] The acceptability of adult male circumcision among MSM in the U.S. has not been reported. The decision to be circumcised would likely be influenced by a range of actual and perceived benefits and risks of circumcision–many of which are not associated with HIV infection. Neonatal circumcision has been shown to reduce the risk of penile cancer, [9] acquisition or transmission of sexually transmitted diseases (STDs), [10], [11], [12] urinary tract infections in infants and young boys, [10], [11] and cervical cancer in female sex partners. [13] The risks of circumcision include surgical complications and sexual dysfunction.[14], [15] The effect of circumcision on sexual pleasure is uncertain. One study of medically indicated circumcision showed overall sexual satisfaction significantly increased, despite a significant increase in erectile dysfunction among some men.[15]

We report the results of a survey conducted among convenience samples of MSM attending gay pride events in the United States assessing their willingness to be circumcised as adults and their perceptions of the benefits and risks of circumcision.

## Methods

We surveyed men attending four community gay pride events (Birmingham, AL; Anchorage, AK; Raleigh-Durham, NC; and Springdale, UT) and three minority gay pride events (Chicago, IL; Charlotte, NC; and St. Louis, MO) in 2006. The minority gay pride events were comprised of two black gay pride events (Charlotte, NC; and St. Louis, MO) and one event that was not specifically targeted to a particular minority group, but was largely attended by blacks (Chicago, IL). We considered our sample a convenience sample, but took additional steps to decrease selection bias. Interviewers were placed throughout the event and approached every *n*th attendee (*n* ranging from 1 to 10, depending on the size of the event) who crossed an imaginary line in order to ask them to complete a brief eligibility screen. At times, MSM were also selected if they approached the interviewers or testing locations. Attendees at gay pride events were eligible to participate in the survey if they were ≥18 years old, born male, and currently identified as male. In addition, attendees at minority gay pride events were eligible if they self-identified as being a member of a racial or ethnic minority. Informed consent was collected verbally from all respondents. Eligible attendees were invited to complete a 10–15 minute survey. Non-monetary incentives (valued at $5 or less) were provided to respondents who completed at least a portion of the survey at three events. The project was determined to be a programmatic evaluation of efforts to implement rapid HIV testing and behavioral assessments among men attending gay pride events, not a research activity. Therefore, review by the Institutional Review Board at the Centers for Disease Control and Prevention (CDC) was not required.

We conducted interviewer-administered surveys with hand-held computers. We used Questionnaire Development System software (QDS™ version 2.4 Nova Research Company, Bethesda, MD) to develop the survey and to collect and manage survey data. We collected data on demographic characteristics and behaviors, circumcision status, and, among uncircumcised MSM, perceived risks and benefits of circumcision and willingness to be circumcised as an adult if scientific studies demonstrated that circumcision reduces the risk of HIV infection among MSM. We asked all respondents: “Are you circumcised (cut), meaning part or the entire foreskin of your penis has been surgically removed?” We showed respondents a flashcard with pictures of both fully circumcised and uncircumcised penises to reduce misclassification of circumcision status. We then asked uncircumcised MSM to rate their agreement or disagreement with a series of statements that used a Likert scale for responses (values: 1 = strongly disagree, 2 = disagree, 3 = neither agree nor disagree, 4 = agree, 5 = strongly agree) to assess their perceptions about risks of adult circumcision (pain, bleeding, or infection of the penis after the surgery) and benefits of adult circumcision (increased sexual pleasure, increased personal hygiene, reduced risk of penile cancer, and reduced risk of sexually transmitted diseases). We dichotomized the scaled responses into agree (neither agree nor disagree, agree, or strongly agree) or disagree (disagree or strongly disagree). We asked uncircumcised respondents the following question and asked them to rate their willingness on a Likert scale (values: 1 = very unlikely, 2 = unlikely, 3 = somewhat likely, 4 = likely, 5 = very likely), “If scientific studies in the United States among men who have sex with men showed that circumcision reduced the risk of HIV infection, would you be willing to be circumcised as an adult?” Preliminary analyses of willingness as an ordinal outcome variable with 5 levels in logistic regression indicated that ordinal regression violated the assumption of proportional odds; therefore we chose to analyze the dichotomized outcome, classified as willing (somewhat likely, likely, or very likely) and unwilling (unlikely or very unlikely).

We restricted the analysis to respondents who did not report that they were HIV-positive and who either identified as homosexual or bisexual or who reported having had sex with a male partner in the past 12 months. We used SAS software version 9.1 (SAS Institute, Cary, NC) to perform statistical analyses. We used the Wilcoxon rank-sum test to determine if median age values were different between groups. We calculated exact odds ratios and 95% confidence intervals and Cochran-Mantel-Haenszel Chi-square tests to determine, in bivariate analyses, if demographic and risk characteristics were significantly associated with: 1) circumcision status among all respondents; and 2) willingness to be circumcised as an adult among uncircumcised respondents. In addition, among uncircumcised respondents, we compared the perceptions of risks and benefits of circumcision by willingness to be circumcised. We limited this analysis to respondents who provided complete data on the perceived risks and benefits of circumcision.

The multivariate analysis included uncircumcised respondents who provided complete data. The preliminary model included covariates that were significantly associated (*p*<0.05) with willingness to be circumcised in the bivariate analysis (race and drug use), as well as the perceived risks and benefits of circumcision. Models were adjusted for age (dichotomized by median age: <33 years old and ≥33 years old). We performed stepwise selection to remove covariates that were not significantly associated with willingness to be circumcised in the multivariate model, and required that covariates have a *p*-value of <0.05 to remain in the multivariate model.[16] Finally, we refit the selected model using exact statistical methods.

## Results

### Circumcision status

We approached 1457 men at the seven gay pride events. Of these, 1127 (77%) men agreed to be surveyed. Respondents at the three sites offering incentives were more likely to agree to be surveyed (p<0.0001). Among sites using incentives, 55% of respondents accepted the survey, compared to 45% in sites that did not use an incentive. A total of 1050 (93%) men consented and were eligible to participate, and 914 (81%) men met our definition of MSM. We excluded 100 (11%) respondents because they were HIV-positive; an additional 31 (3%) respondents were excluded due to missing demographic or risk characteristics. We report findings on 780 (75%) eligible survey respondents who met our definition of MSM, who were not HIV-positive by self-report, and who provided complete survey data. Of the 780 MSM, 133 (17%) reported that they were uncircumcised (Table 1).The median age of uncircumcised and circumcised MSM did not differ (32 years, range: 18–70 years; 32 years, range: 18–68 years, respectively) (*p* = 0.8). Non-white MSM (including blacks, Hispanics, American Indians/Alaskan Natives, Asians/Native Hawaiians/Pacific Islanders and other races) were more likely to be uncircumcised than white MSM. MSM without health insurance were more likely to be uncircumcised. Uncircumcised and circumcised MSM did not differ in level of education attained, number of male sex partners in the past 12 months, proportion reporting unprotected sex with male partners in the past 12 months, use of non-injection or injection drugs in the past 12 months, use of HIV prevention services in the past 12 months, HIV testing history, or self-reported HIV status (HIV-negative vs. unknown HIV status).

**Table 1:** Demographic characteristics, behavioral risk factors, HIV testing history, and use of prevention services among 780 men who have sex with men who attended Gay Pride and Minority Gay Pride events in 7 U.S. cities by circumcision status – 2006.

Characteristic	Total	Uncircumcised		Circumcised		Unadjusted Odds Ratio (95% CI)	Overall *P*-value
	N	n	(%)	n	(%)		
**Total**	780	133	(17)	647	(83)		
**Age in years**							
18–24 years	225	40	(18)	185	(82)	0.7 (0.3–1.3)	
25–34 years	205	30	(15)	175	(85)	0.5 (0.3–1.0)	
35–39 years	125	20	(16)	105	(84)	0.6 (0.3–1.2)	
40–49 years	149	24	(16)	125	(84)	0.6 (0.3–1.2)	
50+ years	76	19	(25)	57	(75)	Referent	0.36
**Race** *							
Hispanic	43	16	(37)	27	(63)	**5.0 (2.3–10.5)**	
Black, Non-Hispanic	198	43	(22)	155	(78)	**2.3 (1.5–3.8)**	
Other†	103	28	(27)	75	(73)	**3.2 (1.8–5.5)**	
White, Non-Hispanic	436	46	(11)	390	(89)	Referent	**<0.0001**
**Health insurance**							
No	202	45	(22)	157	(78)	**1.6 (1.0–2.4)**	
Yes	578	88	(15)	490	(85)	Referent	**0.03**
**Education**							
≤High school	28	8	(29)	20	(71)	2.0 (0.7–4.9)	
>High school	752	125	(17)	627	(83)	Referent	0.12
**Sexual orientation**							
Homosexual	673	112	(17)	561	(83)	0.8 (0.5–1.4)	
Bisexual	107	21	(20)	86	(80)	Referent	0.49
**Risk**							
**Number of male sex partners in the past 12 months** ‡							
0	124	16	(13)	108	(87)	Referent	
1	290	52	(18)	238	(82)	1.5 (0.8–2.7)	
2–5	272	51	(19)	221	(81)	1.6 (0.8–2.9)	
6–10	94	14	(15)	80	(85)	1.2 (0.5–2.6)	0.48
**Any unprotected anal sex in the past 12 months**							
Yes	350	52	(15)	298	(85)	0.8 (0.5–1.1)	
No	430	81	(19)	349	(81)	Referent	0.15
**Non-injection drug use in the past 12 months**							
Yes	207	28	(13)	179	(86)	0.7 (0.4–1.1)	
* Before/during sex*	*112*	*12*	*(11)*	*100*	*(89)*	_	
No	573	105	(18)	468	(82)	Referent	0.13
**Injection drug use**							
Yes	14	1	(7)	13	(93)	0.4 (0.01–2.5)	
No	766	132	(17)	634	(83)	Referent	0.48
**Use of prevention services**							
**Received condoms in the past 12 months** §							
No	168	25	(15)	143	(85)	0.8 (0.5–1.3)	
Yes	609	108	(18)	501	(82)	Referent	0.45
**Ever tested for HIV**							
No	86	20	(23)	66	(77)	1.6 (0.9–2.7)	
Yes	694	113	(16)	581	(84)	Referent	0.13
**Result of most recent HIV test (n = 694)**							
HIV-negative	672	107	(16)	565	(84)	_	
Unknown	22	6	(27)	16	(73)	_	_

* Respondents could select more than one race.

† Other race includes American Indians/Alaskan Native, Asians/Native Hawaiians/Pacific Islanders, and people reporting “other” race.

‡ Two respondents reported >10 male partners.

§ For this question, n = 777 because 3 respondents answered ‘don't know.’

### Willingness to be circumcised

Of the 132 uncircumcised MSM, 71 (53%) indicated that they would be willing to be circumcised (Table 2). In bivariate analyses, black MSM were more likely to be willing to be circumcised than white MSM. MSM who reported using non-injection drugs in the 12 months before the interview were more likely to be willing to be circumcised than MSM who did not report using non-injection drugs.

**Table 2:** Demographic characteristics, behavioral risk factors, HIV testing history, and use of prevention services and agreement with perceived risks and benefits of circumcision and willingness to be circumcised among 133 uncircumcised men who have sex with men not known to be HIV-infected who attended Gay Pride and Minority Gay Pride events in 7 U.S. cities by willingness to be circumcised as an adult – 2006.

Characteristic	Total	Willing		Unwilling		Unadjusted Odds Ratio, (95% CI)	Overall *P*-values
	N	n	%	n	%		
**Total**	133	71	(53)	62	(47)		
**Age in years**							
18–24 years	40	24	(60)	16	(40)	2.0 (0.6–7.3)	
25–34 years	30	15	(50)	15	(50)	1.4 (0.4–5.2)	
35–39 years	20	12	(60)	8	(40)	2.0 (0.5–8.9)	
40–49 years	24	12	(50)	12	(50)	1.4 (0.3–5.5)	
50+ years	19	8	(42)	11	(58)	Referent	0.70
**Race** *							
Hispanic	16	9	(56)	7	(44)	1.8 (0.5–6.8)	
Black, Non-Hispanic	43	30	(70)	13	(30)	**3.2 (1.3–8.7)**	
Other †	28	13	(46)	15	(54)	1.2 (0.4–3.5)	
White, Non- Hispanic	46	19	(41)	27	(59)	Referent	**0.04**
**Health insurance**							
No	45	25	(57)	20	(44)	1.1 (0.5–2.5)	
Yes	88	46	(52)	42	(48)	Referent	0.85
**Education**							
<High school	8	6	(75)	2	(25)	2.8 (0.5–28.8)	
≥High school	125	65	(52)	60	(48)	Referent	0.28
**Sexual orientation**							
Homosexual	112	58	(52)	54	(48)	0.7 (0.2–1.9)	
Bisexual	21	13	(62)	8	(38)	Referent	0.48
**Risk**							
**Number of male sex partners in the past 12 months**							
0	16	6	(38)	10	(63)	Referent	0.81
1	52	32	(62)	20	(38)	2.6 (0.7–10.3)	
2–5	51	27	(53)	24	(47)	1.9 (0.5–7.2)	
6–10	14	6	(43)	8	(57)	1.2 (0.2–6.8)	
**Any unprotected anal sex in the past 12 months**							
Yes	52	24	(46)	28	(54)	0.6 (0.3–1.3)	
No	81	47	(58)	34	(42)	Referent	0.21
**Non-injection drug use in the past 12 months**							
Yes	28	22	(79)	6	(21)	**4.1 (1.5–13.5)**	
Before/during sex	12	9	(75)	3	(25)	_	
No	105	49	(47)	56	(53)	Referent	**0.003**
**Use of prevention services**							
**Received condoms in the past 12 months**							
No	25	10	(40)	15	(60)	1.9 (0.7–5.3)	
Yes	108	61	(56)	47	(44)	Referent	0.18
**Ever tested for HIV**							
No	20	11	(55)	9	(45)	0.9 (0.3–2.7)	
Yes	113	60	(53)	53	(47)	Referent	1.00
**Result of most recent HIV test (n = 113)**							
HIV-negative	107	55	(51)	52	(49)	-	
Unknown	6	5	(83)	1	(17)	-	_
**Concerned because it might be painful** ‡							
Strongly agree	46	21	(32)	25	(41)		
Agree	37	21	(32)	16	(26)		
Neither agree nor disagree	14	8	(12)	6	(10)		
Disagree	11	8	(12)	3	(5)		
Strongly disagree	19	8	(12)	11	(18)		0.75
**Concerned because it might cause postoperative bleeding** ‡							
Strongly agree	29	14	(21)	15	(25)		
Agree	41	25	(38)	16	(26)		
Neither agree nor disagree	18	11	(17)	7	(11)		
Disagree	22	11	(17)	11	(18)		
Strongly disagree	17	5	(8)	12	(20)		0.21
**Concerned because it might cause postoperative infection** ‡							
Strongly agree	28	15	(23)	13	(21)		
Agree	40	24	(36)	16	(26)		
Neither agree nor disagree	22	9	(14)	13	(21)		
Disagree	21	11	(17)	10	(16)		
Strongly disagree	16	7	(11)	9	(15)		0.38
**Consider because it might increase sexual pleasure** ‡							
Strongly agree	4	2	(3)	2	(3)		
Agree	20	15	(23)	5	(8)		
Neither agree nor disagree	19	14	(21)	5	(8)		
Disagree	43	21	(32)	22	(36)		
Strongly disagree	41	14	(21)	27	(44)		**<0.01**
**Consider because it might increase personal hygiene** ‡							
Strongly agree	9	3	(5)	6	(10)		
Agree	25	20	(30)	5	(8)		
Neither agree nor disagree	16	11	(17)	5	(8)		
Disagree	46	20	(30)	26	(43)		
Strongly disagree	31	12	(18)	19	(31)		**<0.03**
**Consider because it might reduce risk of penile cancer** ‡							
Strongly agree	9	6	(9)	3	(5)		
Agree	30	26	(39)	4	(7)		
Neither agree nor disagree	31	16	(24)	15	(25)		
Disagree	37	12	(18)	25	(41)		
Strongly disagree	20	6	(9)	14	(23)		**<0.0001**
**Consider because it might reduce risk of STDs** ‡							
Strongly agree	9	8	(12)	1	(2)		
Agree	31	24	(36)	7	(11)		
Neither agree nor disagree	17	10	(15)	7	(11)		
Disagree	39	11	(17)	28	(46)		
Strongly disagree	31	13	(20)	18	(30)		**<0.0001**

* Respondents could select more than one race.

† Other race includes American Indians/Alaskan Native, Asians/Native Hawaiians/Pacific Islanders, and people reporting “other” race.

‡ Five uncircumcised respondents did not provide data for the perceived risks and benefits statements, therefore N = 127.

The majority (59–64%) of respondents agreed or strongly agreed with statements about the perceived risks of adult circumcision, such as postoperative pain, bleeding and infection (Table 2). In bivariate analyses when agreement was dichotomized into “agree” (strongly agree, agree and neither agree nor disagree) and “disagree” (strongly disagree and disagree) categories, there were no differences between MSM who agreed with these statements and those who did not by willingness to be circumcised. A smaller proportion of MSM (26–48%) agreed or strongly agreed with statements about the perceived benefits of circumcision. However, MSM who were willing to be circumcised were more likely to report that they agreed that circumcision might increase sexual pleasure (OR: 3.6**;** 95% CI: 1.5–8.8), increase hygiene (OR: 3.0; 95% CI: 1.3–6.8), reduce risk of penile cancer (OR: 4.7; 95% CI: 2.1–10.8), and reduce risk of STDs (OR: 5.3; 95% CI: 2.3–12.5). When respondents with “neither agree nor disagree” responses were dropped from the analysis, the findings did not change appreciably.

### Results of the multivariate analysis

In the age-adjusted multivariate model among 127 uncircumcised MSM who provided complete data on the perceived risks and benefits of circumcision, black MSM (OR: 3.4; 95% CI: 1.3–9.8) were more likely than MSM of all other races to be willing to be circumcised as an adult (Table 3). MSM who reported non-injection drug use in the past 12 months (OR: 6.1; 95% CI: 1.8–23.7) were more likely to be willing to be circumcised as an adult than MSM who did not report non-injection drug use. In addition, MSM who agreed that reduced risk of penile cancer was a benefit of circumcision were more likely to be willing to be circumcised as an adult (OR: 4.7; 95% CI: 2.0–11.9) compared to men who disagreed with this statement. When respondents with “neither agree nor disagree” responses were dropped from the analysis, black MSM (5.4; 95% CI: 1.7–19.0), MSM who reported non-injection drug use in the past 12 months (8.9; 95% CI: 2.2–47.6), and MSM who agreed that reduced risk of penile cancer was a benefit of circumcision (7.5; 95% CI: 2.5–25.1) were more likely to be willing to be circumcised as an adult.

**Table 3:** Willingness to be circumcised among 127 uncircumcised men who have sex with men not known to be HIV-infected who attended Gay Pride and Minority Gay Pride events in 7 U.S. cities – 2006.

Characteristic	Adjusted Odds Ratio, 95% Confidence Interval	P-value
**Age**		
<33 years *	2.0 (0.8–4.9)	0.16
≥33 years	Referent	
**Race**		
Non-Hispanic black race	**3.4 (1.3–9.8)**	**<0.01**
All other races	Referent	
**Non-injection drug use**		
Yes	**6.1 (1.8–23.7)**	**<0.01**
No	Referent	
**Willing to consider circumcision to reduce risk of penile cancer**		
Yes	**4.7 (2.0–11.9)**	**<0.0001**
No	Referent	

* Median age of respondents was 33 years.

## Discussion

More than 80% of the MSM interviewed at seven recent gay pride and minority gay pride events reported that they were circumcised. The circumcision prevalence we report among our respondents reflects national trends. Using data from the National Health and Nutrition Examination Survey (NHANES), Xu and colleagues found that 79% of men born between 1940 and 1990 were circumcised.[17] They found that circumcision prevalence was at its lowest in men born in 1940–1949 (71%), increased and remained stable from 1950–1979 at about 81% and then decreased slightly in the 1980s (78%). Overall, from 1940–1990, differences in national trends existed by race, with a circumcision prevalence of 73% among black men compared to 88% among white men. Among black men, the prevalence of circumcision dramatically increased from 50% in men born in the 1940s to 91% to men born in the 1970s. Like all other men in this study, circumcision rates decreased to 81% for black men born in the 1980s. Because the majority of men are circumcised at birth in the United States, the potential of circumcision as a prevention intervention in the United States may be limited.

Over half of the uncircumcised respondents in this analysis indicated that they would be willing to be circumcised as adults, and blacks were three times more likely to be willing than whites. Although blacks make up less than 13% of the U. S. population, they account for approximately half of Americans currently estimated to be living with HIV/AIDS and among men living with HIV/AIDS, 47% are black. [4] In a recent meta-analysis examining the increased risk of HIV infection among black MSM, the authors found that black MSM are no more likely to engage in sexual risk behaviors, including unprotected anal intercourse, than other MSM.[18] Yet disparities in HIV infection rates among black MSM and other MSM persist. Since black men are less likely to be circumcised shortly after birth, and are more willing to be circumcised as adults, there exists an opportunity to reduce disparities in HIV infection rates if circumcision is found to reduce HIV infection among MSM in the U.S.

The finding that black MSM were more likely to report willingness to be circumcised was somewhat unexpected, because black MSM may be less likely to trust health-care professionals and the government, based on a history of racism within this country and misconduct in research, as evidenced in the Tuskegee syphilis study.[19] In addition, black heterosexual men and MSM are more likely to endorse conspiracy beliefs, such as the belief that HIV does not cause AIDS and that HIV is a man-made virus.[20], [21] Considering this, messages tailored to specific racial/ethnic groups of MSM may be more successful in addressing perceived risks and benefits of circumcision.

We examined risk behaviors because we thought that MSM might be more willing to be circumcised if they thought that a one-time biomedical intervention could reduce their risk of acquiring HIV without changing their risks behaviors. MSM reporting non-injection drug use in the past 12 months were more likely than MSM who did not use non-injection drugs to report that they would be willing to be circumcised as an adult. MSM who report non-injection drug use may see circumcision as a method to reduce their risk of HIV infection without changing their drug use or prevention behaviors. Because the data collected in this project do not allow us to determine why persons who use non-injection drugs may be more willing to be circumcised as adults, further investigation of this association is warranted. For example, it would be important to clarify whether non-injection drug use was a marker for greater perception of risk of HIV infection, which may have in turn motivated interest in a biomedical prevention intervention.

Our respondents were more likely to endorse concerns about circumcision than they were to endorse motivators to be circumcised; however only agreement with the belief that adult circumcision may reduce the risk of penile cancer was associated with willingness to be circumcised in multivariate analysis. Interestingly, our results parallel those of surveys of uncircumcised Kenyan men, of whom 64% reported preferring to be circumcised due to beliefs that circumcision would decrease risk of penile cancer, decrease risk of acquiring STD and HIV/AIDS, or increase sexual pleasure.[22] It is important to note, however, that neonatal circumcision has been shown to reduce risk of penile cancer, but that adult circumcision does not appear to provide similar benefits and may increase risk for penile cancer if conducted for the purpose of treating other penile conditions.[23], [24] Therefore, it is unclear whether adult circumcision will prevent penile cancer. Discussion of the potential benefits and risks of adult circumcision, and particular attention to the perceptions that some MSM may have about its benefits and risks should be included in educational materials if circumcision is ever recommended for MSM in the United States.

This analysis has several limitations. Respondents were selected in a way that minimized bias, but our respondents constituted a convenience sample and respondents receiving an incentive were more likely to agree to be interviewed than those who did not. Therefore, respondents are not representative of all MSM who attended these events. The seven gay pride events were located in low- to mid-level HIV prevalence areas, limiting their generalizability to MSM who live in areas with higher prevalence. In addition, MSM at these events are more likely to be comfortable with disclosing their sexual identity to others and therefore may have different risks and attitudes and/or beliefs about issues related to sex than MSM who do not attend such public events. Circumcision status was obtained by self-report, which may be less accurate than ascertaining circumcision status by physical examination, although a recent study shows high concordance between self-report and actual circumcision status. [25] In addition, we attempted to prevent misclassification by using flashcards with pictures of circumcised and uncircumcised penises. Participants were asked to provide perceptions to only a limited number of possible benefits and concerns about adult circumcision, and we may have not captured information about other issues that might be important to MSM in making decisions about adult circumcision. In addition, because overall willingness to be circumcised was not assessed separately from willingness to be circumcised if scientific evidence demonstrated a reduced risk of HIV acquisition, we were not able to compare whether the decision to be circumcised would be influenced by the evidence to support the effectiveness of circumcision in the reduction in risk of HIV acquisition. Furthermore, we did not assess trust in scientific evidence in general, which may also influence respondents' willingness to be circumcised if scientific evidence demonstrated a reduced risk of HIV acquisition. Understanding that circumcision would not obviate the need to maintain safe sex practices might reduce the willingness of some MSM to agree to adult circumcision. Furthermore, this analysis addressed questions involving respondents' future intentions, which are imperfect predictors of future behavior, but may provide an insight into whether adult circumcision would be considered feasible with this population.

Although no randomized clinical trials to evaluate the protective effect of circumcision have been planned for MSM in the U.S., discussions about public health campaigns to promote circumcision are ongoing. It is important to keep in mind that circumcision must be offered in combination with other HIV prevention efforts. It is also important to understand how sexual risk behaviors may change after adult circumcision occurs. For example, some MSM in this sample may have indicated a willingness to be circumcised because they thought it would reduce their risk for HIV infection and that they would no longer need to use condoms. Although circumcision would be only one component of a comprehensive risk reduction plan, there may be confusion about the benefits that this intervention can offer. We did not give respondents background information that circumcision would provide only partial protection against HIV and that condom use would still be recommended after the procedure. MSM who are circumcised might, in fact, decrease their condom use or have more sexual partners because they believe that they no longer need to use condoms after being circumcised.[26] If circumcision programs were implemented for MSM, they would need to include educational messages that informed potentially interested adult MSM that this procedure alone would not be enough to prevent HIV infection. Future studies should determine whether MSM would be willing to be circumcised if they knew that condom use would still be necessary to ensure optimal protection against HIV even after circumcision. In addition, further qualitative studies should be conducted to examine the perceived risks and benefits of circumcision among MSM. Lastly, black MSM have higher rates of HIV infection and demonstrate more willingness to be circumcised than white MSM. Future studies should examine willingness and perceptions of circumcision to determine what might increase willingness to be circumcised among other MSM of color. Despite these complexities, we have shown that the majority of uncircumcised MSM may be willing to be circumcised as adults if this was recommended as an HIV prevention intervention in the United States.
